# Inhibition of human cytomegalovirus replication by interferon alpha can involve multiple anti-viral factors

**DOI:** 10.1099/jgv.0.001929

**Published:** 2023-12-08

**Authors:** Shabab Chowdhury, Katie A. Latham, Andy C. Tran, Christopher J. Carroll, Richard J. Stanton, Michael P. Weekes, Stuart J. D. Neil, Chad M. Swanson, Blair L. Strang

**Affiliations:** ^1^​ Institute of Infection & Immunity, St George’s, University of London, London, UK; ^2^​ Institute of Molecular & Cellular Sciences, St George’s, University of London, London, UK; ^3^​ Division of Infection and Immunity, Cardiff University School of Medicine, Cardiff, UK; ^4^​ Cambridge Institute for Medical Research, School of Clinical Medicine, University of Cambridge, Cambridge, UK; ^5^​ Department of Infectious Diseases, School of Immunology & Microbial Sciences, King’s College London, London, UK

**Keywords:** human cytomegalovirus interferon alpha PML ZAP

## Abstract

The shortcomings of current direct-acting anti-viral therapy against human cytomegalovirus (HCMV) has led to interest in host-directed therapy. Here we re-examine the use of interferon proteins to inhibit HCMV replication utilizing both high and low passage strains of HCMV. Pre-treatment of cells with interferon alpha (IFNα) was required for robust and prolonged inhibition of both low and high passage HCMV strains, with no obvious toxicity, and was associated with an increased anti-viral state in HCMV-infected cells. Pre-treatment of cells with IFNα led to poor expression of HCMV immediate-early proteins from both high and low passage strains, which was associated with the presence of the anti-viral factor SUMO-PML. Inhibition of HCMV replication in the presence of IFNα involving ZAP proteins was HCMV strain-dependent, wherein a high passage HCMV strain was obviously restricted by ZAP and a low passage strain was not. This suggested that strain-specific combinations of anti-viral factors were involved in inhibition of HCMV replication in the presence of IFNα. Overall, this work further supports the development of strategies involving IFNα that may be useful to inhibit HCMV replication and highlights the complexity of the anti-viral response to HCMV in the presence of IFNα.

## Introduction

The betaherpesvirus human cytomegalovirus (HCMV) remains a notable cause of human morbidity and mortality worldwide [1]. While many different vaccine candidates are in development, there is no widely available vaccine against HCMV [1]. Those direct-acting anti-HCMV drugs currently in clinical use have many shortcomings, including drug resistance and toxicity [1–3].

Recently developed approaches to inhibiting HCMV replication have focused on host-directed therapy, which positively or negatively influences cellular factors involved in HCMV replication [3]. Prominent examples of these strategies include the use of artemisinin compounds and kinase inhibitors that inhibit cellular factors required for HCMV replication [3]. However, other areas of host-directed therapy remain largely unexplored and/or require re-examination. This includes exploring the use of different interferon proteins to inhibit HCMV replication, as there may be multiple interferon proteins capable of inhibiting HCMV replication. Plus, there are aspects of interferon action on HCMV replication which have not been recently revisited, such as the possible reversible inhibition of HCMV replication in the presence and absence of interferon [4].

Exposure of cells to interferon proteins results in an intracellular anti-viral state, which comes about by interaction of interferon proteins with cell surface receptors, leading to an intracellular signalling cascade, which promotes expression of multiple anti-viral proteins [5]. Arguably, the type I interferon response, stimulated by interferon α or β proteins, is the most widely studied interferon response against HCMV.

Inhibition of HCMV replication by type I interferon proteins has a long history, with early reports suggesting that interferon could not inhibit HCMV replication [6]. However, this may have been the result of low concentrations of type I interferon proteins used in the study [6]. More recent studies have strongly indicated that the type I interferon response is inhibitory to HCMV replication. This data includes a report where HCMV secretion was inhibited upon administration of IFNα to patients [7], several reports indicating that pre-treatment of cells before infection *in vitro* with type I interferon proteins restricted HCMV replication [4, 8–11], and the observation that inhibition of type I interferon signalling allows greater replication of HCMV *in vitro* [12]. Additionally, numerous reports of HCMV-encoded mechanisms to antagonize interferon production, antagonize interferon signalling or antagonize the function of anti-viral proteins expressed in response to interferon emphasize that the type I interferon response is a barrier to HCMV replication [13–15].

However, it is not well understood how the presence of type I interferon proteins affects the molecular mechanisms of HCMV replication, in particular, the ability of different HCMV strains to evade inhibition by the type I interferon response. We have recently demonstrated that a low passage strain of HCMV whose genome content is similar HCMV clinical isolates (strain Merlin) was able to efficiently inhibit the production of the anti-viral factor zinc finger anti-viral protein (ZAP) in response to type I interferon, whereas a high passage laboratory strain commonly used in laboratory experiments and containing a genomic deletion plus several mutations (strain AD169) could not [16]. Comparison of how these different HCMV strains might replicate in the presence of type I interferon proteins and how this might relate to the action of anti-viral factors (such as ZAP) has not, to our knowledge, been performed.

HCMV interaction with interferon proteins beyond the type I response also requires further exploration. For example, the interaction of HCMV with the type III interferon response. Although type I (IFNα/β) and III (IFNλ) interferon proteins utilize different receptor complexes to activate intracellular signalling, the repertoires of anti-viral proteins produced by each response is broadly similar [5]. These repertoires include proteins such as ZAP [17], that we and others have demonstrated are anti-HCMV proteins [16, 18, 19]. This suggests that, like IFNα/β, IFNλ proteins could inhibit HCMV replication. This is supported by reports that certain gene alleles associated with expression of IFNλ proteins can positively or negatively affect control of HCMV replication *in vivo* [20–22] and a report that treatment of intestinal cells with IFNλ3 could limit HCMV protein production [23]. HCMV replication in the presence of IFNλ3 was not assessed in the aforementioned study [23].

Additionally, there may be long-standing observations about the interaction of HCMV and interferons that may need to be revisited to understand how those proteins can be used in a therapeutic setting. A previous study examining treatment of HCMV-infected human fibroblast cell cultures from foreskin biopsies with type I interferon indicated that inhibition of HCMV replication was reversible [4]. Similar observations have been made upon infection of murine cells, including fibroblastic murine cells, with a betaherpesvirus related to HCMV; murine cytomegalovirus (MCMV) [24–26]. In those experiments the presence of type I interferon could promote MCMV latency [24–26]. Together, these data suggest reversible inhibition of virus replication by type I interferon is a common feature of betaherpesvirus replication. To our knowledge, the reversible effects of type I interferon on HCMV replication has not been revisited since [4].

As we have previously suggested that there may be differences in the ability of HCMV strains to inhibit responses to IFNα [16], we set out to test the ability of a high passage laboratory strain (AD169) and a low passage strain Merlin(R1111), similar to wild-type HCMV, to replicate in the presence of a IFNα or IFNλ3. While IFNλ3 had very little effect on HCMV replication, we observed robust and prolonged inhibition of HCMV replication upon pre-treatment of cells with IFNα before HCMV infection, with no obvious toxicity. This inhibition of HCMV replication was associated with loss of immediate early protein expression and the presence of the known anti-viral protein PML-SUMO. Consistent with our previous observations [16], the role of ZAP in inhibiting HCMV replication was strain-dependent; wherein ZAP-inhibited replication of AD169, but Merlin considerably less so. Our observations emphasize that multiple anti-viral proteins, including ZAP, are likely responsible for inhibition of HCMV replication in the presence of IFNα.

## Methods

### Cells and viruses

Human foreskin fibroblast (HFF) cells (clone Hs27) were obtained from American Type Culture Collection, no. CRL-1634 (ATCC, Manassas, VA). Bulk populations of HFF cells containing CRISPR inhibiting expression of either Luciferase or all ZAP isoforms have been previously described [16]. All cells were maintained in complete media: Dulbecco’s Modified Eagle’s Medium (DMEM) (Gibco) containing 10 % (v/v) fetal bovine serum (FBS) (Gibco), plus 1 % mixture of penicillin and streptomycin.

HCMV strain AD169 was a gift from Donald Coen (Harvard Medical School). The generation of HCMV strain Merlin(R1111), which contains mutations in genes RL13 and UL128 to allow release of cell-free virus, from a bacmid has been reported previously [27]. AD169 virus expressing green fluorescent protein (GFP) from an ectopic site in the AD169 genome under control of the HCMV major immediate early (IE) promoter (AD169-GFP) was generated from a bacmid as described in [28]. Merlin(R2582), a virus derived from Merlin(R1111) that expresses a fusion peptide of the HCMV IE protein UL36 and GFP separated by a self-cleaving P2A protein sequence, has also been previously described [29]. In all cases, titres were determined by serial dilution of viral supernatant onto HFF monolayers, which were then covered in DMEM containing 5 % (v/v) FBS, antibiotics and 0.6 % (w/v) methylcellulose. After incubation for 14 days, cells were stained with crystal violet and plaques in the infected cell monolayers were counted. Titre was expressed as p.f.u. ml^−1^.

### Viral yield reduction assays

HFF cells were plated at 5×10^4^ cells per well in 24-well plates. After overnight incubation, cells were either infected with 5×10^4^ p.f.u. of HCMV or incubated for a further 24 h in the presence or absence of interferon or drug without infection. After this further 24 h period, cells incubated in the presence or absence of interferon or drug without infection were infected with 5×10^4^ p.f.u. of HCMV. HCMV viruses used in each experiment are indicated in the text and figure legends. In each case, after virus adsorption for 1 h at 37 °C, cells were washed and incubated with 0.5 ml of media in the presence or absence of interferons or drug throughout virus replication. Infected cells were incubated for 96 h at 37 °C before supernatant was removed from cells for analysis of virus titre by plaque counting, as described in the previous section. Data showing virus production over time is shown in Fig. S1A, available in the online version of this article.

### Interferons and Ruxolitinib

Interferon-α [INTRON A (interferon alfa-2b), Merck] was a kind gift from Steve Goodbourn (St George’s, University of London). Interferon-λ3 was purchased from Bio-Techne. Both interferon proteins were resuspended in complete cell-culture media. Ruxolitinib was purchased from Cambridge Bioscience and resuspended in dimethyl sulfoxide (DMSO). In all experiments cells were treated with 1000 U ml^−1^ of interferon-α (or the equivalent volume of complete cell culture), 100 ng ml^−1^ interferon-λ3 (or the equivalent volume of complete cell culture) or 10 µM Ruxolitinib (or the equivalent volume of DMSO). The final concentration of DMSO in all experiments was maintained at <1 % (v/v). In all experiments, cells were treated with the concentrations of interferons mentioned above as these were the concentrations at which maximum inhibition of HCMV replication was observed. Use of higher concentrations of interferons did not yield greater inhibition of HCMV replication (Fig. S1B and S1C).

### Western blotting

Conditions under which HFF or HFF CRISPR cells were infected are detailed in the text and figure legends. Lysate of uninfected or infected cells were prepared for Western blotting by washing the cells once in PBS (SIGMA), suspending the cells directly in 2×Laemmli buffer containing 5 %β−mercaptoethanol, and incubating at 95 °C for 5 min.

Proteins were separated on 8 % or 10 % (v/v) polyacrylamide gels and transferred to a Hybond-ECL membrane (Amersham Biosciences) using a semi-dry protein transfer apparatus. The membranes were blocked at room temperature for at least 90 min using TBS containing 0.1 % Tween-20 and 5 % dried powdered milk (TBSTM) and then incubated overnight at 4 °C in TBSTM plus primary antibodies: antibodies recognizing HCMV IE1/2, UL57, or pp28, (all Virusys, 1 : 1000 dilution), β-actin (SIGMA, 1 : 5000 dilution), ZAP (Abcam, ab154680, 1 : 5000 dilution, recognizing all ZAP isoforms), MxA, STAT1-Tyr701p (both Cell Signalling, no. 43575, 1 : 1000 dilution), PML (Bethyl, A301-167A, 1 : 1000 dilution), MRPS39 (PTCD3) (ProteinTech, 25158–1-AP, 1 : 1000 dilution) and TOM20 (ProteinTech, 11802–1-AP, 1 : 1000 dilution).

After incubation in TBSTM with primary antibodies, the membrane was washed extensively with TBST and incubated for 60 min at room temperature with TBSTM containing anti-mouse- or anti-rabbit-horseradish peroxidase (HRP) conjugated antibodies (Millipore and Cell Signalling Technologies, respectively), to detect primary antibodies. After further washing with TBST and TBS, chemiluminescence solution (GE Healthcare) were used to detect secondary antibodies on x-ray film (GE Healthcare).

Where indicated in the text, relative band intensity (band intensity relative to β-actin signal in the same lane) was analysed using ImageJ software, obtained from the NIH (USA).

### Cell number and viability (MTT) assays

To count cells, HFF cells were seeded at high or low concentrations cells per well into 24-well plates. High numbers of cells (5×10^4^ cells per well) were to assess cell viability, whereas low numbers of cells (5×10^3^ cells per well) were to assess both cell viability and cell proliferation. After overnight incubation to allow cell attachment, cells were treated for 96 h with IFNα or left untreated. Cells were removed from wells using trypsin, resuspended in complete cell media and counted using a Countess Automated Cell Counter (Invitrogen) using the manufacturer’s instructions.

In MTT assays, HFF were seeded at high (5×10^3^ cells per well) or low (5×10^2^ cells per well) numbers cells per well into 96-well plates. After overnight incubation to allow cell attachment, cells were treated for 96 h with IFNα or left untreated. MTT assays were carried out on cells in the wells of 96-well plates according to the manufacturer’s instructions (GE Healthcare). The ability of cellular NAD(P)H-dependent cellular oxidoreductase enzymes to reduce the tetrazolium dye 3-(4,5-dimethylthiazol-2-yl)−2,5-diphenyltetrazolium bromide (MTT) to formazan was measured in colorimetric assay, read on a FLUOstar Omega Microplate Reader.

### Flow-cytometry analysis

Cells were infected with GFP expressing viruses as described in the text and figure legends. At 24 h post-infection, uninfected and infected cells were trypsinized, washed once in PBS, and then resuspended in PBS. GFP expression in cells was analysed using flow cytometry. In each case, 10 000 cells were acquired using a Beckman Coulter CytoFLEX S cytometer. Data were analysed using FlowJo V10 to determine number of GFP positive cells and mean fluorescent intensity of GFP positive cells.

## Results

### Pre-treatment of HFF cells with IFNα robustly inhibited replication of HCMV

**Fig. 1.**
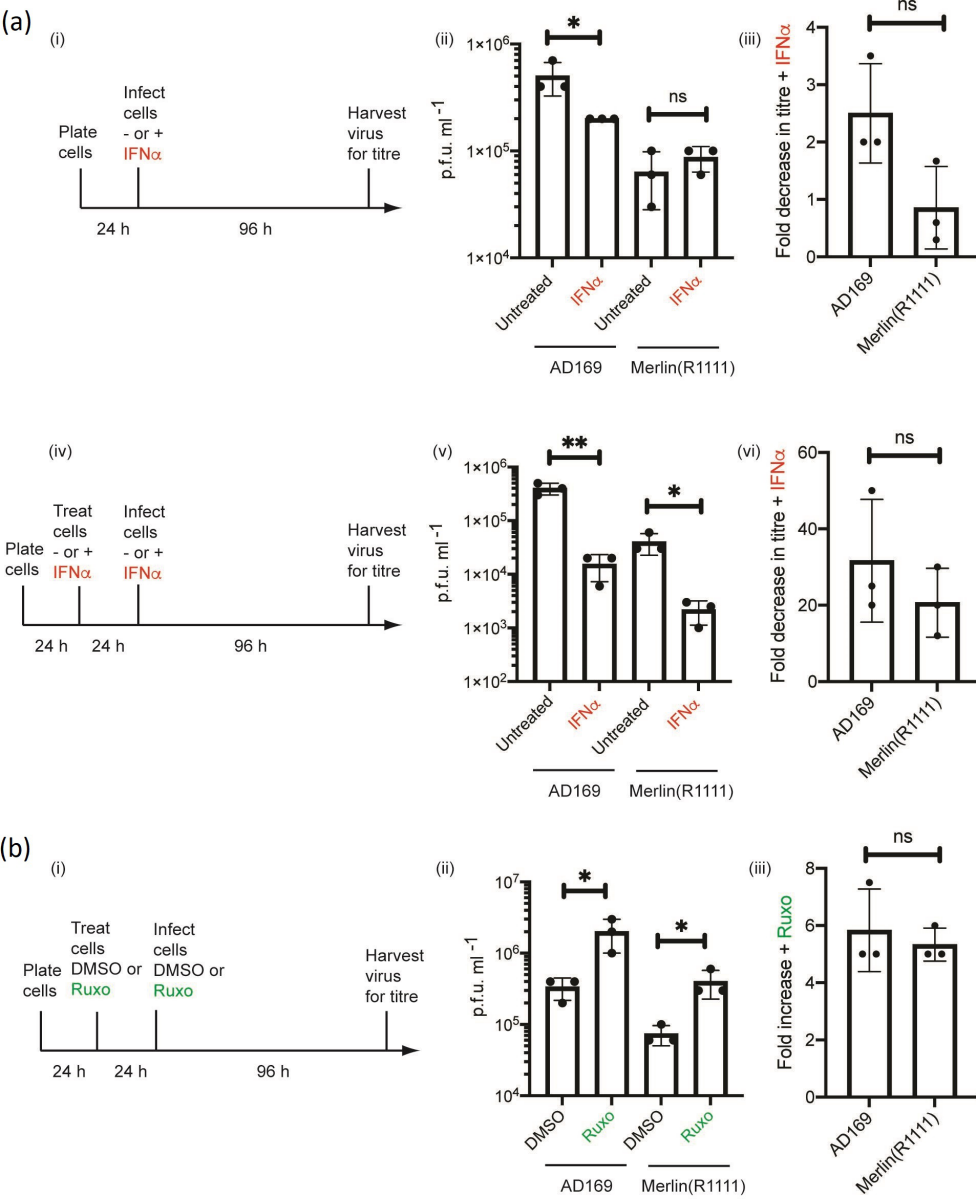
HCMV replication in HFF cells treated with IFNα or Ruxolitinib. (**a**) (**i**) and (iv) Diagram of experiments: HFF cells were treated with IFNα at the time of infection (or left untreated) or pre-treated for 24 h with IFNα (or left untreated) and then infected in the presence and absence of IFNα. Treatment of cells continued throughout infection with either AD169 or Merlin(R1111) for 96 h. (ii) and (**v**) Titre in p.f.u. ml^−1^ of each experiment. (iii) and (iv) Fold decrease in HCMV titre in the presence of IFNα compared to HCMV titre from infected untreated cells. (**b**) (**i**) Diagram of experiments: HFF cells were treated with Ruxolitinib (Ruxo) or the equivalent volume of DMSO at the time of infection or pre-treated with Ruxo or the equivalent volume of DMSO then infected in the presence of either Ruxo or DMSO. Treatment of cells continued throughout infection with either AD169 or Merlin(R1111). (ii) Titre in p.f.u. ml^−1^ of each experiment. (iii) Fold increase in HCMV titre in the presence of Ruxo compared to HCMV titre from infected cells treated with DMSO. In each figure data is representative of three independent experiments (black data points) and presented as the average (block) and standard deviation (error bars) of the data. In each experiment statistical relevance was examined using Student's *t*-test. ns=not significant (ns), *P*=<0.05 (*,**).

We compared inhibition of AD169 and Merlin(R1111) replication when IFNα was added to human foreskin fibroblast (HFF) cells at the time of infection or when HFF cells were pre-treated with IFNα before infection [Fig. 1a(i) and (iv), respectively]. Addition of IFNα at the time of infection had a modest effect on AD169 replication, but no obvious impact on Merlin(R1111) replication [Fig. 1a(ii) and (iii)]. However, pre-treatment of HFF cells with IFNα before infection resulted in robust inhibition of both AD169 and Merlin(R1111) replication [Fig. 1a (v) and (vi)], indicating that setting an anti-viral state within HFF cells before infection was required for robust inhibition of replication of both HCMV strains.

To confirm that inhibition of type I interferon signalling before infection could influence HCMV replication, in the absence of exogenous IFNα HFF cells were also pre-treated with the Janus-kinase inhibitor Ruxolitinib [Fig. 1b(i)], which inhibits intracellular signalling from the type 1 interferon receptor. Treatment of HFF cells with Ruxolitinib resulted in increased replication of both AD169 and Merlin(R1111) [Fig. 1b(ii) and (iii)], confirming that type 1 interferon signalling could inhibit replication of both HCMV strains.

**Fig. 2.**
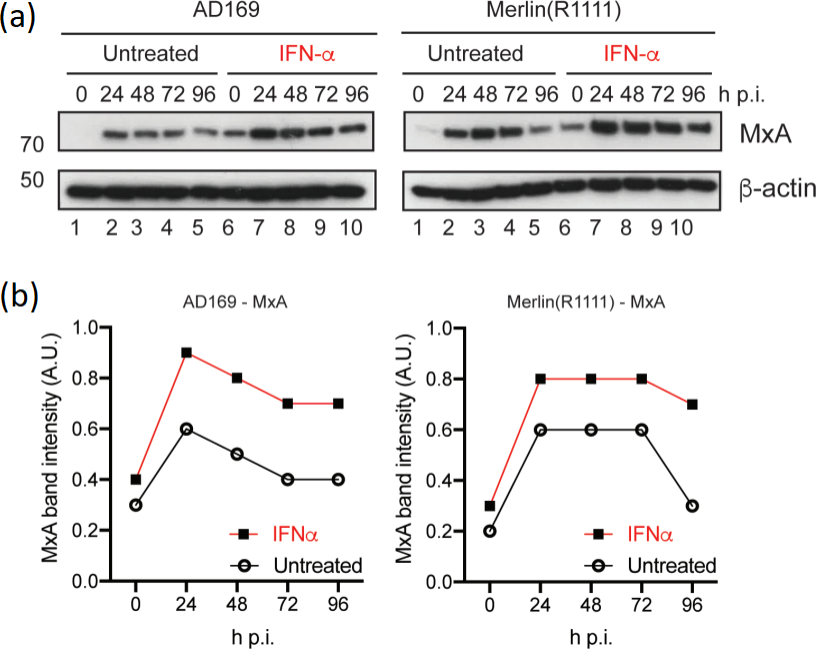
MxA expression in the presence and absence of IFNα. HFF cells were pre-treated for 24 h with IFNα (or left untreated) and then infected in the presence and absence of IFNα. Treatment of cells continued throughout infection with either AD169 or Merlin(R1111). HFF cell lysates were prepared for Western blotting at the time points indicated in the figure [hours post-infection (h p.i.)]. Uninfected HFF cell lysates were treated or untreated IFNα were also prepared for Western blotting at the time of infection (0 h p.i.). (**a**) Western blotting. Proteins recognized by the antibodies used in the experiment are indicated to the right of the figure. The positions of molecular weight markers (kDa) are indicated to the left of the figure. (**b**) Quantification of Western blotting. Relative band intensity [band intensity of MxA relative to β-actin signal in the same lane, arbitrary units (A.U.)] was analysed using ImageJ using data from two independent experiments. The mean of each data point from those experiments is shown. Hours post-infection (h p.i.).

We also analysed how pre-treatment of HFF cells with IFNα before infection stimulated an anti-viral state. We analysed expression of MxA, a canonical protein expressed in response to type 1 interferon proteins, in the presence and absence of IFNα (Fig. 2). The presence of IFNα stimulated MxA expression in uninfected HFF cells. In AD169 and Merlin(R1111) infected HFF cells pre-treated with IFNα, MxA expression was greater than in HFF cells not pre-treated with IFNα. This increased anti-viral state in infected HFF cells pre-treated with IFNα largely persisted over the course of infection in HFF cells infected with both HCMV strains, which could explain why robust inhibition of both AD169 and Merlin(R1111) was observed in Fig. 1.

### Treatment with IFNα did not cause overt cellular cytotoxicity

As shown by many small molecule inhibitors of HCMV replication [3], anti-viral activity can be due to cellular cytotoxicity of compounds, not an anti-viral effect. Therefore, to ensure that inhibition of HCMV replication by AD169 and Merlin(R1111) seen in Fig. 1 was not due to cellular cytotoxicity caused by exposure of cells to IFNa, we analysed cell division (Fig. S2A) and cellular cytotoxicity (Fig. S2B) in uninfected cells exposed to IFNα. These assays were carried out using a high number of HFF cells (similar to that used in Figs 1 and 2) to examine cell viability and a low number of HFF cells to examine both cell viability and cell proliferation of IFNα-treated cells.

Treatment of HFF cells with IFNα did not result in any reduction in cell numbers using high or low numbers of cells (Fig. S2A). However, when cells were analysed with a cytotoxicity assay that measures mitochondrial activity (MTT assay), treatment of HFF cells with IFNα resulted in a modest deficit in cell viability at low numbers of cells (Fig. S2B). When HFF cells were analysed for expression of mitochondrial proteins (mitochondrial ribosome protein MRPS39 and mitochondrial membrane protein TOM20), no difference in the expression of mitochondrial proteins was found in the presence and absence of IFNα (Fig. S2C). Therefore, the deficit in mitochondrial activity in the presence of IFNα at low cell concentrations was not due to a deficit in the number of mitochondria in those conditions. Overall, these data indicated that exposure of cells to IFNα did not cause obvious cytotoxicity and, therefore, indicated that the inhibition of HCMV replication seen in Fig. 1 was unlikely to be due to cytotoxic effects of IFNα on cells. However, IFNα could have a very modest effect on HFF cell viability under certain conditions, which may have had a minor influence on the ability of HFF cell cultures to support HCMV replication.

### Robust inhibition of HCMV replication by IFNα, but not IFNλ3

Before examining the action of IFNα in greater detail, we also compared the ability of IFNα and IFNλ3 to inhibit HCMV replication. As in Fig. 1, pre-treatment of HFF cells with IFNα resulted in robust inhibition of AD169 and Merlin(R1111) replication [Figs. S3A(i) and S3A(ii)]. Pre-treatment of HFF cells with IFNλ3 resulted in a modest, but not statistically significant, inhibition of either HCMV strain [Fig. S3A(i) and S3A(iii)]. Consistent with these observations, we observed obvious differences in the ability of IFNα and IFNλ3 to stimulate phosphorylation of STAT1 (STAT-Tyr701p, which occurs on activation of both the type I and type III interferon receptors) and the expression of MxA (Fig. S3B). Thus, the differences in the ability of the different interferon proteins to inhibit HCMV replication likely reflected their abilities to simulate an anti-viral state in HFF cells. In this experimental setting only IFNα could robustly inhibit HCMV replication.

### Inhibition of HCMV replication was associated with inhibition of HCMV entry and protein expression

**Fig. 3.**
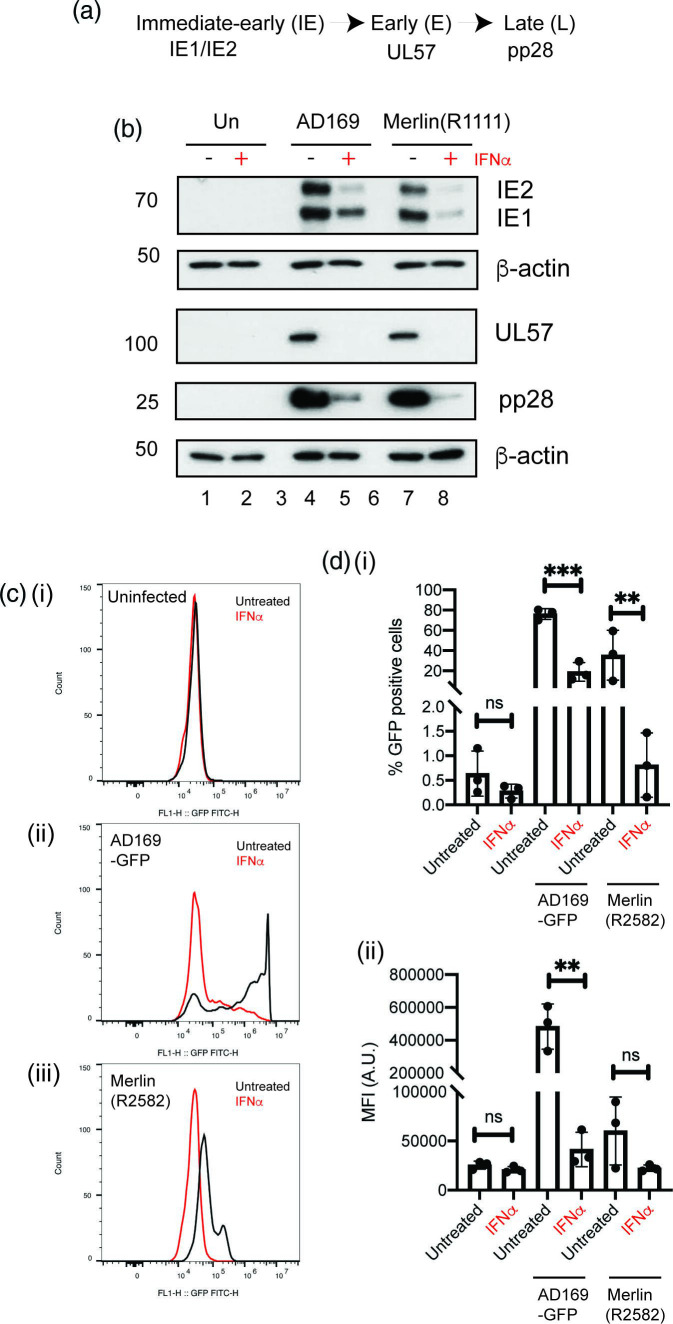
Western blotting and FACS analysis of HCMV infection in the presence and absence of IFNα. (**a**) Schematic of HCMV protein expression with relevant proteins grouped into kinetic classes. (**b**) HFF cells were pre-treated for 24 h with IFNα (or left untreated) and then infected in the presence and absence of IFNα. Treatment of cells continued throughout infection with either AD169 or Merlin(R1111). Cell lysates were prepared for Western blotting at 96 h post-infection (h p.i.). Uninfected cell lysates were treated or untreated IFNa were also prepared for Western blotting at the time of infection (0 h p.i.). Proteins recognized by the antibodies used in the experiment are indicated to the right of the figure and the positions of molecular weight markers (kDa) are indicated to the left of the figure. (**c and d**) HFF cells were pre-treated for 24 h with IFNα (or left untreated) and then infected in the presence and absence of IFNα. Treatment of cells continued throughout infection with AD169 or Merlin viruses expressing GFP. At 24 h post-infection cells were analysed using flow cytometry. (**c**) Flow-cytometry data from a representative experiment. (**d**) (**i**) number of cells expressing GFP (ii) mean fluorescent intensity of GFP expressing cells. Data is representative of three independent experiments (black data points) and presented as average (block) and standard deviation (error bars) of the data. Statistical relevance was examined using Student's *t*-test. ns=not significant (ns), *P*=<0.05 (*,**,***).

To understand how IFNα inhibited HCMV replication, we examined expression of proteins produced from each class of HCMV transcription (Fig. 3a). Pre-treatment of HFF cells with IFNα resulted in poor protein expression from each class (IE1/2, UL57 and pp28) in both AD169 and Merlin(R1111)-infected HFF cells (Fig. 3b). Poor UL57 and pp28 expression was likely the result of poor immediate-early protein (IE1/IE2) expression. IFNα inhibited IE protein production at all times points tested during AD169 and Merlin(R1111) replication (Fig. S1C).

We then sought to understand if poor IE1/2 expression in the presence of IFNα was due to a defect in HCMV entry into the cell or due to lack of protein expression in infected cells. Therefore, we assayed the ability of HCMV viruses expressing green fluorescent protein (GFP) [AD169-GFP and Merlin(R2582)] [28, 29] to infect cells. We reasoned that if the number of cells expressing GFP in the presence and absence of IFNα was similar, IFNα did not inhibit HCMV entry into the cell. Furthermore, we reasoned that if the number of cells expressing GFP was similar in the presence and absence of IFNα, but the fluorescence intensity of GFP expressing cells decreased in the presence of IFNα, then pre-treatment of cells with IFNα did not inhibit HCMV entry into the cell, but did inhibit protein expression from the HCMV genome.

We observed that pre-treatment of HFF cells with IFNα resulted in a decrease in both the number of cells expressing GFP and the fluorescence intensity of GFP in infected cells (Fig. 3c, d), albeit the effect of IFNα on Merlin(R2582) was very modest in one experimental replicate, affecting the overall statistical significance of the data (Fig. 3d). Overall, however, we interpreted the data in Fig. 3d as pre-treatment of HFF cells with IFNα possibly led to both inhibition of HCMV entry into the cell and inhibition of HCMV protein expression in the infected cell.

### Inhibition of HCMV replication was associated with the presence of PML-SUMO proteins

We reasoned that inhibition of HCMV replication was caused by anti-viral proteins expressed in the presence of IFNα. A range of anti-viral proteins expressed in response to type I interferon proteins have anti-HCMV activity [18]. To our knowledge, none of these proteins are known to inhibit HCMV entry into the cell. Therefore, we focused on examining anti-viral proteins expressed in the presence of IFNα that could inhibit IE protein production.

The first anti-viral barrier to replication that the HCMV genome likely encounters in the nucleus are PML bodies. Proteins in PML bodies, including PML, come into contact with incoming HCMV genomes and form structures that can contribute to inhibition of HCMV transcription [30], including transcriptional inhibition of the alternatively spliced HCMV mRNA that encodes IE1 and IE2. Key to the anti-HCMV function of PML is the post-translational addition of SUMO proteins to PML (PML-SUMO) [31]. Antagonism of PML SUMOylation by HCMV IE1 antagonizes the anti-viral effects of PML bodies [31]. It has been demonstrated that transcription of mRNA encoding PML and other anti-viral proteins in PML bodies is stimulated by the presence of IFNβ during HCMV infection [32].

**Fig. 4.**
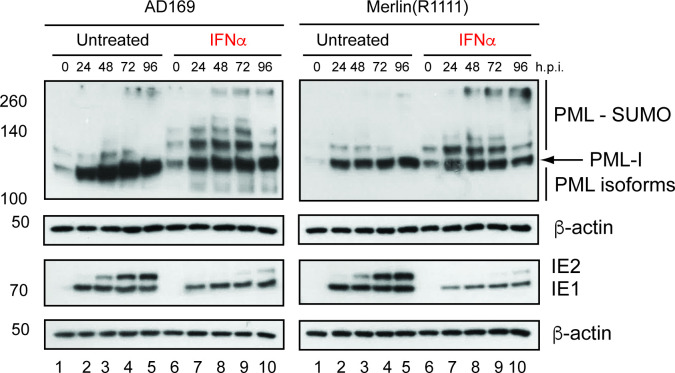
Western blotting of HCMV and PML proteins expressed in the presence and absence of IFNα. HFF cells were pre-treated for 24 h with IFNα (or left untreated) and then infected in the presence and absence of IFNα. Treatment of cells continued throughout infection with either AD169 or Merlin(R1111). Cell lysates were prepared for Western blotting at the time points indicated in the figure [hours post-infection (h p.i.)]. Uninfected cell lysates were treated or untreated IFNa were also prepared for Western blotting at the time of infection (0 h p.i.). Proteins recognized by the antibodies used in the experiment are indicated to the right of the figure and the positions of molecular weight markers (kDa) are indicated to the left of the figure.

As in Fig. 3, pre-treatment of HFF cells with IFNα led to loss of IE1 and IE2 expression. Expression of PML isoforms can vary between different cell lines, as can the molecular weights of those isoforms [31, 33, 34]. Here we observed expression of PML forms in our HFF cell line similar to those previously reported for another HFF cell line [34]. In the absence of virus or IFNα a version of PML [isoform I (PML-I)] was detectable at approximately 120 kDa, as could at least one high molecular weight SUMOylated form of PML-I, but smaller molecular weight isoforms of PML could not be detected (Fig. 4). In the presence of virus without IFNα treatment, PML-I expression increased, but expression of SUMOylated PML-I proteins increased less so (Fig. 4), consistent with antagonism of PML SUMOylation by IE1 [31]. However, upon treatment with IFNα high molecular weight forms of SUMOylated PML-I could be observed in HCMV infected cells (Fig. 4). Therefore, a combination of both IFNα treatment and HCMV replication led to an anti-viral state associated with the presence of PML-SUMO proteins in both AD169 and Merlin(R1111) in infected HFF cells. As PML-SUMO was present in uninfected HFF cells treated with IFNα, it is possible that expression of IE1 from incoming HCMV genomes was not sufficient to overcome the block to HCMV replication by PML-SUMO proteins already present in the cell. Therefore, the presence of PML-SUMO in infected HFF cells treated with IFNα was associated with poor immediate-early protein expression.

### Inhibition of HCMV replication in the presence of IFNα by ZAP was strain-dependent

Anti-viral proteins other than PML expressed in the presence of IFNα (IDO1, RIPK2) can inhibit HCMV replication [35, 36] and may have been involved in inhibition of HCMV replication that we observed here. Additionally, we and others have recently observed that ZAP proteins can inhibit HCMV replication, including the ZAP isoform whose expression is stimulated by IFNα, ZAP-S [16, 18, 19]. Additionally, we found that inhibition of HCMV replication by ZAP was strain-dependent, as AD169 replication was restricted by ZAP but Merlin(R1111) replication was not [16]. These phenotypes were associated with downregulation of ZAP-S in Merlin(R1111)-infected cells, but not AD169-infected cells [16]. As we did not see strain-dependent differences in resistance to IFNa in IFNα pre-treated cells, we investigated ZAP expression in cells pre-treated with IFNα.

**Fig. 5.**
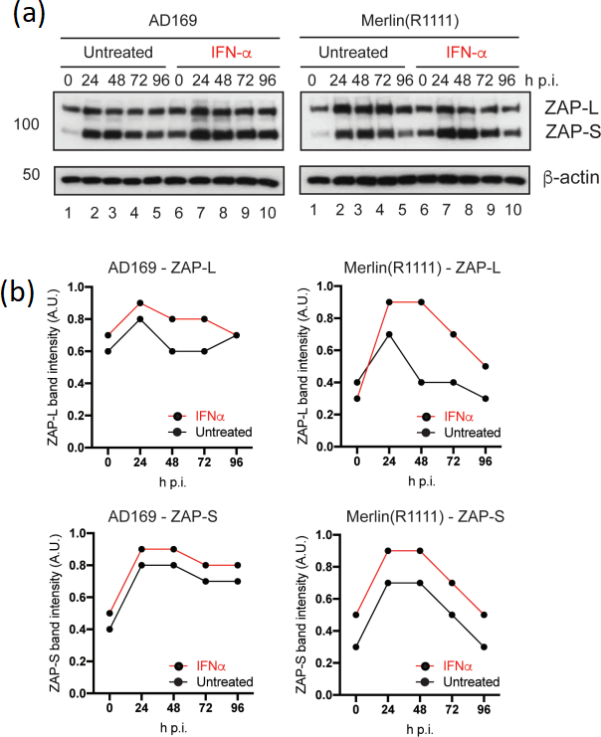
ZAP isoform expression in the presence and absence of IFNα. HFF cells were pre-treated for 24 h with IFNα (or left untreated) and then infected in the presence and absence of IFNα. Treatment of cells continued throughout infection with either AD169 or Merlin(R1111). HFF cell lysates were prepared for Western blotting at the time points indicated in the figure [hours post-infection (h p.i.)]. Uninfected HFF cell lysates were treated or untreated IFNα were also prepared for Western blotting at the time of infection (0 h p.i.). (**a**) Western blotting. Proteins recognized by the antibodies used in the experiment are indicated to the right of the figure (large and small ZAP isoforms, ZAP-L and ZAP-S, respectively). The positions of molecular weight markers (kDa) are indicated to the left of the figure. (**b**) Quantification of Western blotting. Relative band intensity [band intensity of each ZAP isoform relative to β-actin signal in the same lane, arbitrary units (A.U.)] was analysed using ImageJ using data from two independent experiments. Hours post-infection (h p.i.). The mean of each data point from those experiments is shown.

We analysed ZAP isoform expression in the presence and absence of IFNα. Pre-treatment of HFF cells with IFNα resulted in expression of ZAP-S in uninfected cells (Fig. 5a). Upon infection, pre-treatment of HFF cells with IFNα resulted in greater expression of both ZAP-L and ZAP-S in both AD169 and Merlin(R1111)-infected HFF cells (Fig. 5). However, consistent with our previous observations [16], we observed an obvious decrease in ZAP-S expression over time in Merlin(R1111), but not AD169, infected HFF cells in the presence and absence of IFNα (Fig. 5). However, similar to expression of MxA (Fig. 2), we found that expression of both ZAP isoforms was greater in the presence of IFNα, suggesting that although ZAP-S expression obviously decreased in Merlin(R1111)-infected cells, there was sufficient ZAP-S to inhibit Merlin(R1111) replication in HFF cells pre-treated IFNα.

**Fig. 6.**
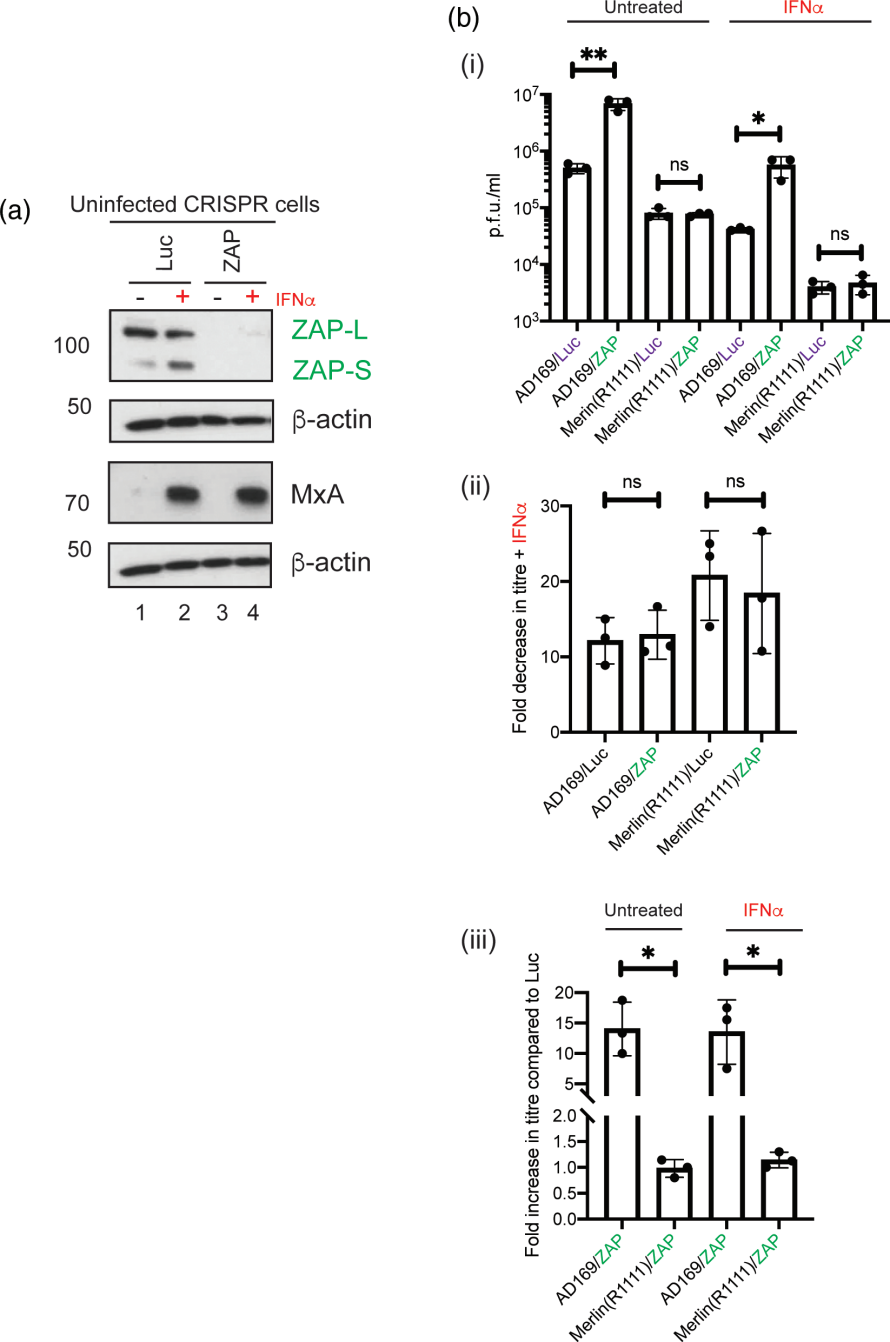
HCMV replication in CRISPR containing cells in the presence and absence of IFNα. (**a**) Uninfected HFF CRISPR-Luc (Luciferase) or CRISPR-ZAP HFF cells were treated with IFNα or left untreated.Cell lysates were prepared for Western blotting 24 h post-treatment. Proteins recognized by the antibodies used in the experiment are indicated to the right of the figure. The positions of molecular weight markers (kDa) are indicated to the left of the figure. (**b**) (**i**) HFF CRISPR-Luc or CRISPR-ZAP cells were pre-treated for 24 h with IFNα (or left untreated) and then infected in the presence and absence of IFNα. Treatment of cells continued throughout infection with either AD169 or Merlin(R1111) for 96 h. Titre in p.f.u. ml^−1^ of each experiment was calculated. Data is representative of three independent experiments (black data points) and presented as average (block) and standard deviation (error bars) of the data. Statistical relevance was examined using Student's *t*-test. ns=not significant (ns), *P*=<0.05 (*,**). (ii) Fold decrease in HCMV titre in the presence of IFNα compared to HCMV titre from infected untreated cells. (iii) Fold increase in HCMV titre in CRISPR-ZAP cells compared to CRISPR-Luc cells.

We then assayed HCMV replication in the presence and absence of ZAP isoforms. Using bulk populations of HFF cells containing CRISPR that inhibited the expression of both ZAP isoforms (ZAP-L and ZAP-S) [16], we confirmed that the loss of ZAP expression did not compromise the ability of IFNα to set an anti-viral state [37], as similar MxA expression was seen in the presence and absence of ZAP proteins in IFNα-treated cells (Fig. 6a). Additionally, this data confirmed the loss of ZAP isoform expression in HFF cells.

Upon examination HFF cells infected with AD169 or Merlin(R1111) that did or did not express ZAP isoforms, we found that pre-treatment of HFF cells with IFNα before infection resulted in a decrease in HCMV replication in all conditions tested [Fig. 6b(i) and (ii)]. However, in agreement with our previous observations [16], we found that in the presence and absence of IFNα loss of ZAP proteins could increase AD169, but not Merlin(R1111), replication [Fig. 6b(ii) and (iii)]. As in our previous work, we interpret this strain-dependent inhibition of HCMV replication as the ability of Merlin(R1111), but not AD169, to control ZAP expression, in particular ZAP-S [16]. Thus, although ZAP isoform expression was greater in the presence of IFNα (Fig. 5a), the decrease in ZAP-S expression observed (Fig. 5a) may have been sufficient to prevent inhibition of Merlin(R1111) replication by ZAP.

In summary, IFNα could inhibit replication of both AD169 and Merlin(R1111), but in the presence of IFNα ZAP was an inhibitor of AD169, but not Merlin(R1111). Therefore, there may be strain-dependent combinations of anti-viral proteins inhibiting replication of either AD169 or Merlin(R1111). The combinations of proteins inhibiting either AD169 or Merlin(R1111) replication can include known anti-HCMV proteins expressed in the presence of type I interferon proteins, such as ZAP, PML-SUMO, RIPK2 and IDO [16, 18, 19, 31, 35, 36].

### IFNα had prolonged repressive effects on HCMV replication

It has previously been reported that inhibition of HCMV replication in human fibroblast cultures derived from foreskin biopsies by IFNα was reversible [4], similar to observations made upon infection of several murine cell types with MCMV, including fibroblastic murine cells [24–26]. Reversible inhibition of HCMV replication would not be advantageous to many therapeutic strategies. Therefore, we investigated if the action of IFNα on HCMV replication was reversible, as previously reported [4].

**Fig. 7.**
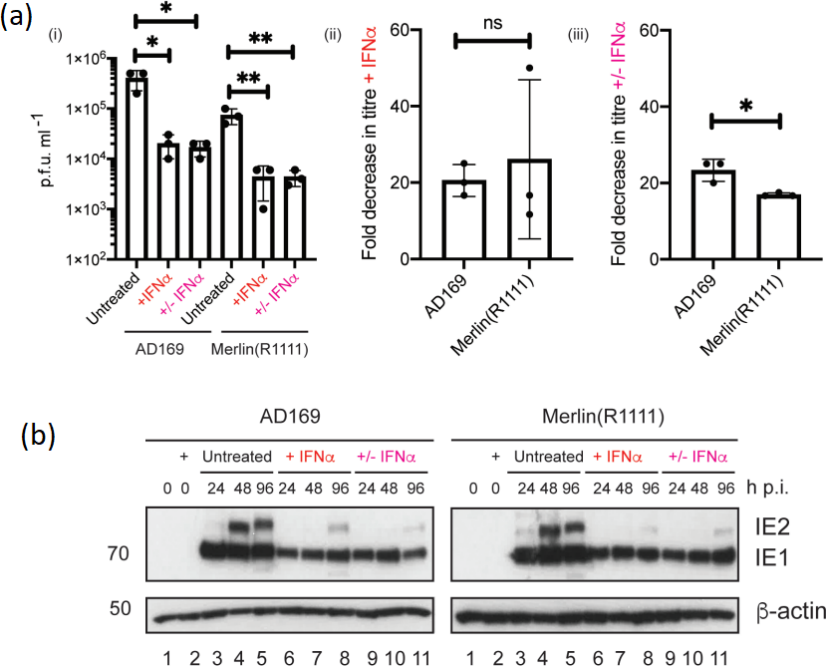
HCMV replication in HFF cells in the continuous and discontinuous presence of IFNα. (**a**) (**i**) HFF cells were pre-treated for 24 h with either IFNαor left untreated and then infected in the presence and absence of IFNα. Treatment of cells with IFNα continued throughout infection with either AD169 or Merlin(R1111) (+IFNα) or was discontinued at the time of infection (+/-IFNα). Titre in p.f.u. ml^−1^ of each experiment at 96 h post-infection was calculated. Data is representative of three independent experiments (black data points) and presented as the average (block) and standard deviation (error bars) of the data. Statistical relevance was examined using Student's *t*-test. ns=not significant (ns), *P*=<0.05 (*,**). (ii) Fold decrease in HCMV titre in the continuous presence of IFNα (+IFNα) compared to HCMV titre from infected untreated cells. (iii) Fold decrease in HCMV titre in the discontinuous presence of IFNα (+/-IFNα) compared to HCMV titre from infected untreated cells. (**b**) HFF cells were treated as in (a) and cell lysates were prepared for Western blotting at the time points indicated in the figure. Proteins recognized by the antibodies used in the experiment are indicated to the right of the figure. The positions of molecular weight markers (kDa) are indicated to the left of the figure.

As above (Fig. 1), pre-treatment of HFF cells with IFNα with continuous treatment of infected HFF cells with IFNα led to robust inhibition of AD169 and Merlin(R1111) replication, which was associated with loss of IE1 and IE2 expression [Fig. 7a(i) and (ii), b]. However, near identical results were found when infected HFF cells were pre-treated with IFNα, but IFNα was not maintained in the cell culture from the time of infection [Fig. 7a(i) and (ii), b]. Therefore, the anti-viral state set by pre-treatment of HFF cells with IFNα could not be overcome by either HCMV strain when IFNα was absent after infection, demonstrating that in these experimental conditions IFNα had prolonged repressive effects on HCMV replication. We attempted to maintain HCMV-infected HFF cells infected with high and low m.o.i.s and treated with IFNα in culture past 7–10 days. However, this resulted in destruction of cell monolayers and no infectious HCMV virus could be detected (data not shown). This suggested that in our hands inhibition of HCMV replication by IFNα may have been irreversible. Consistent with our analysis in the previous sections, these prolonged or irreversible effects could have been caused by a combination of anti-viral factors acting on HCMV.

## Discussion

When IFNα was added to HFF cells at the time of infection replication of the high passage laboratory strain AD169 was modestly inhibited, whereas replication of the low passage HCMV strain Merlin was not. This was consistent with our previous observations that compared to Merlin, AD169 did not effectively control the anti-viral state cause by type I interferons at the time of infection [16, 38], likely due to downregulation of type I interferon signalling proteins in Merlin-infected cells [38]. However, when cells were exposed to IFNα before infection, replication of both HCMV strains was robustly inhibited. Therefore, there were strain-dependent differences in the ability of HCMV to evade the type I interferon response, but this was dependent upon when HCMV encountered the intracellular anti-viral state stimulated by IFNα. This emphasized that if IFNα is to be used in a therapeutic setting it would be advantageous to ensure that it is administered early in infection so that HCMV encounters the intracellular anti-viral state when the virus enters the cell. Additionally, our data suggests that high concentrations of IFNα may be required to efficiently inhibit HCMV replication. Both of these points would make therapeutic use of IFNα challenging and may help explain contradictory findings about the inhibitory effects outlined in the Introduction [6, 7]. However, the apparent prolonged effects of IFNα on HCMV replication may indicate that continual treatment with IFNα is not required.

In this study we examined the ability of IFNα to inhibit replication of HCMV strains that infect cells as cell-free virus. It has been previously demonstrated that replication of cell-associated HCMV Merlin was not obviously inhibited in cells pre-treated with type I interferons [11]. Thus, treatment of HCMV patients using exogenous IFNα may inhibit replication of cell-free virus during HCMV transmission and have less effect on virus dissemination *in vivo*.

In contrast to the robust inhibition of HCMV replication by IFNα, we found a modest, but not statistically significant, inhibition of HCMV replication by IFNλ3. This was likely due to the inability of IFNλ3 to set a robust anti-viral state within cells. This may have been due to weak interactions between IFNλ3 and its cognate cell surface receptor [39] or differences in expression of type I and type III receptors on the cell line used here. To our knowledge, there are no reports testing the ability of IFNλ proteins other than IFNλ3 (IFNλ1, IFNλ2 and IFNλ4) to inhibit HCMV replication. Our data suggests that further examination of interaction between HCMV and IFNλ3 may be of interest in attempts to inhibit HCMV replication. These experiments will likely require development of novel infectious models of HCMV replication involving cell lines that are highly susceptible to activation of an anti-viral state via IFNλ proteins, such as hepatocyte or intestinal cells [39]. Such models do not appear to be available at present. For example, IFNλ3 has been shown to inhibit IE protein expression in a transformed intestinal cell line [23], but transformed cells such as those do not support HCMV replication.

The intrinsic ability of PML bodies to inhibit transcription from the HCMV genome, and their antagonism by the HCMV protein IE1, has been widely studied [40, 41]. The anti-viral role of PML bodies in response to type I interferon proteins has been previously investigated, showing that an increase in mRNA encoding the HCMV inhibitory proteins, including PML, in response to IFNβ produced from cells upon HCMV infection [32]. Here, we expand on those observations [32] by analysing protein expression, showing that while PML protein is expressed in response to IFNα, expression of both PML and PML-SUMO is greatest in HCMV-infected cells treated with IFNα. This overexpression of PML and PML-SUMO that HCMV encounters when it entered the cell and attempted to replicate was most likely a major cause of inhibition of immediate-early protein expression and, therefore, HCMV replication.

However, other factors were likely to be involved in inhibition of HCMV replication in cells treated with IFNα. For example, screens of proteins produced in response to type I interferon signalling indicate that anti-viral factors such as RIPK2 and IDO are likely to inhibit HCMV replication in the conditions we use here [18, 35, 36]. We also investigated the role of ZAP in inhibition of HCMV replication [16, 18, 19]. Consistent with our previous observations [16], we found differences in the ability of AD169 and Merlin(R1111) to evade ZAP function. However, we conclude that the ability of IFNα to inhibit replication of HCMV was likely due to a combination of anti-viral proteins expressed in response to IFNα, including ZAP and SUMO-PML, and there may be strain-dependent differences in anti-viral factors required to inhibit replication of different HCMV strains.

Contrary to a previous report [4], we found that IFNα caused prolonged, or perhaps irreversible, inhibition of HCMV replication under the conditions we used here. In the aforementioned study [4], HCMV replication was undetectable for up to 16 days in the presence of IFNα and then could be detected when IFNα was withdrawn from the culture of fibroblasts derived from foreskin biopsies at 16 days post-infection. It is possible that the differences in our studies are the result of differences in experimental procedure. We speculate that the HFF cell line used in our studies and the primary cell cultures taken from foreskin biopsies used previously [4] may be able to support HCMV replication in different ways. We further speculate that primary cell cultures from foreskin biopsies used previously [4] may have contained as yet unrecognized fibroblastic or monocytic cells capable of supporting IFNα-dependent HCMV latency and reactivation. However, while different conditions for this experiment could be explored, we demonstrate conditions under which inhibition of HCMV replication may be irreversible, which will be useful for considering future therapeutic strategies involving IFNα.

Additionally, our data contrasts with that seen during MCMV infection and suggests that reversible inhibition of virus replication by type I interferons may not be a feature conserved across betaherpesvirus replication. In further contrast, previous reports link the presence of IFNα to the establishment of MCMV latency and the withdrawal of IFNα from MCMV-infected murine cell cultures was required for reactivation of MCMV from latency [24–26], whereas proteins expressed in response to type I interferon proteins are down regulated during HCMV latency in human monocytes [42]. Therefore, the type I interferon response is likely to be detrimental to the establishment of HCMV latency and/or HCMV reactivation from latency. Thus, the two betaherpesvirues have different relationships with the type I interferon response during virus latency. That said, our observations made in this study and aforementioned work on HCMV latency [42] suggest that treatment with IFNα will have inhibitory effects on both productive HCMV replication and HCMV latency/reactivation.

Any future strategy targeting HCMV replication or HCMV latency/reactivation may benefit from utilizing newly developed IFNα mutants that can stimulate an anti-viral response without potently stimulating immunomodulatory or antiproliferative effects that may be harmful *in vivo* [43–46].

## Supplementary Data

Supplementary material 1
