# Supplementary material for: Inhibition of human cytomegalovirus replication by interferon alpha can involve multiple anti-viral factors
Source: J Gen Virol. 2023 Dec 8;104(12):001929. doi: 10.1099/jgv.0.001929 (PMC10770924; doi:10.1099/jgv.0.001929)
Supplement: Supplementary material 1 [file jgv-104-1929-s001.pdf]

Supplementary Data

Inhibition of Human Cytomegalovirus Replication by Interferon Alpha can involve multiple anti-viral factors

Shabab Chowdhury[a], Katie A Latham[a], Andy C Tran[a], Christopher J Carroll[b], Richard J Stanton[c], Michael P Weekes[d], Stuart J D Neil[e], Chad M Swanson[e] & Blair L Strang[a #]

Institute of Infection & Immunity[a], Institute of Molecular & Cellular Sciences[b], St George's, University of London, London, UK; Division of Infection and Immunity, Cardiff University School of Medicine, Cardiff, UK[c]; Cambridge Institute for Medical Research, School of Clinical Medicine, University of Cambridge, Cambridge, UK[d]; Department of Infectious Diseases, School of Immunology & Microbial Sciences, King's College London, London, UK[e].

#Address correspondence to Blair L Strang, bstrang@sgul.ac.uk

Figure S1 Inhibition of HCMV strains in the presence of interferons (A) HFF cells were pre-treated for 24 hours with IFNα, IFNλ3 (or left untreated) and then infected in the presence and absence of interferons. (A) Treatment of cells with 1000 U/ml IFNα continued throughout infection with either AD169 or Merlin(R1111). Titre in plaque forming units/ml (p.f.u./ml) was determined from virus supernatants collected at the time points indicated in the figure. (B and C). Treatment of cells with interferons continued throughout infection with either AD169 or Merlin(R1111) for 96 hours. Concentrations of interferons used are indicated in each figure. Titre in plaque forming units/ml (p.f.u./ml) was determined from virus supernatants collected at the 96 hours post infection. (C) HFF cell lysates were prepared for western blotting at the time points indicated in the figure (hours post infection (h.p.i.)). Uninfected HFF cell lysates were treated or untreated IFNα were also prepared for western blotting at the time of infection (0 h.p.i.). Proteins recognized by the antibodies used in the experiment are indicated to the right of the figure. The positions of molecular weight markers (kDa) are indicated to the left of the figure. AD169 (A), Merlin(R1111) (M).

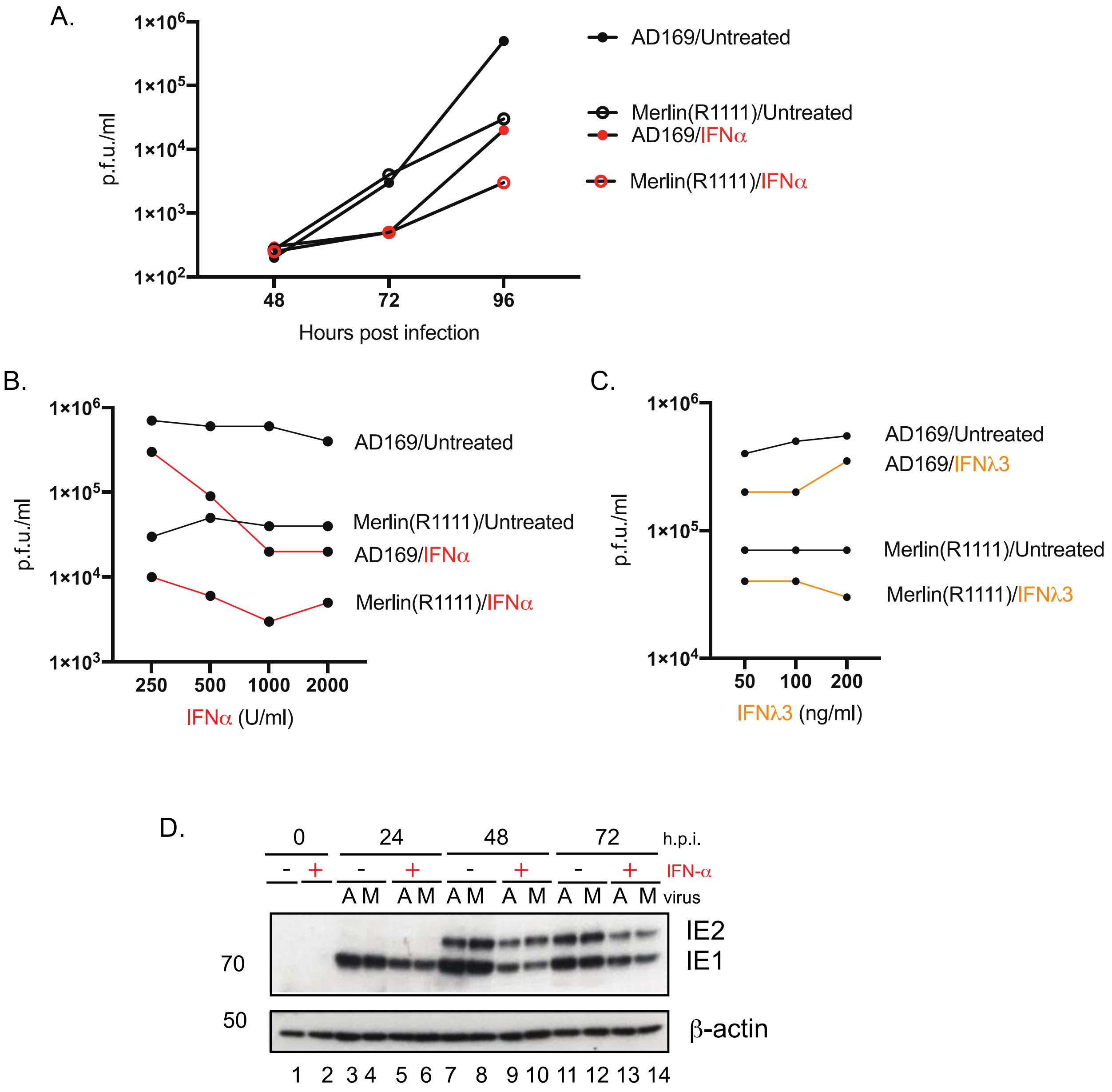
A.
1×10^6
1×10^5
1×10^4
1×10^3
1×10^2
p.f.u./ml
48
72
96
Hours post infection
AD169/Untreated
Merlin(R1111)/Untreated
AD169/IFNα
Merlin(R1111)/IFNα
B.
1×10^6
1×10^5
1×10^4
1×10^3
p.f.u./ml
250
500
1000
2000
IFNα (U/ml)
AD169/Untreated
Merlin(R1111)/Untreated
AD169/IFNα
Merlin(R1111)/IFNα
C.
1×10^6
1×10^5
1×10^4
p.f.u./ml
50
100
200
IFNλ3 (ng/ml)
AD169/Untreated
AD169/IFNλ3
Merlin(R1111)/Untreated
Merlin(R1111)/IFNλ3
D.
0
24
48
72
h.p.i.
- + - + - + - +
IFN-α
A M A M A M A M A M A M
virus
70
50
IE2
IE1
β-actin
1 2 3 4 5 6 7 8 9 10 11 12 13 14

Figure S1

Figure S2 Assessment of cell number and cytotoxicity in the presence and absence of IFNα. (A and B) Uninfected HFF cells plated at (i) high or (ii) low numbers were treated with IFNα or left untreated for 96 hours. Cell number or cell health in each condition was then investigated using cell counting or MTT assays. The percentage of data from IFNα treated cells compared to untreated cells was calculated. In each figure data is representative of three independent experiments (black data points) and presented as average (block) and standard deviation (error bars) of the data. (C) Uninfected HFF cells plated at high or low numbers were treated with IFNα or left untreated for 96 hours. At 96 hours post infection. time samples were prepared for western blotting. Proteins recognized by the antibodies used in the experiment are indicated to the right of the figure. The positions of molecular weight markers (kDa) are indicated to the left of the figure.

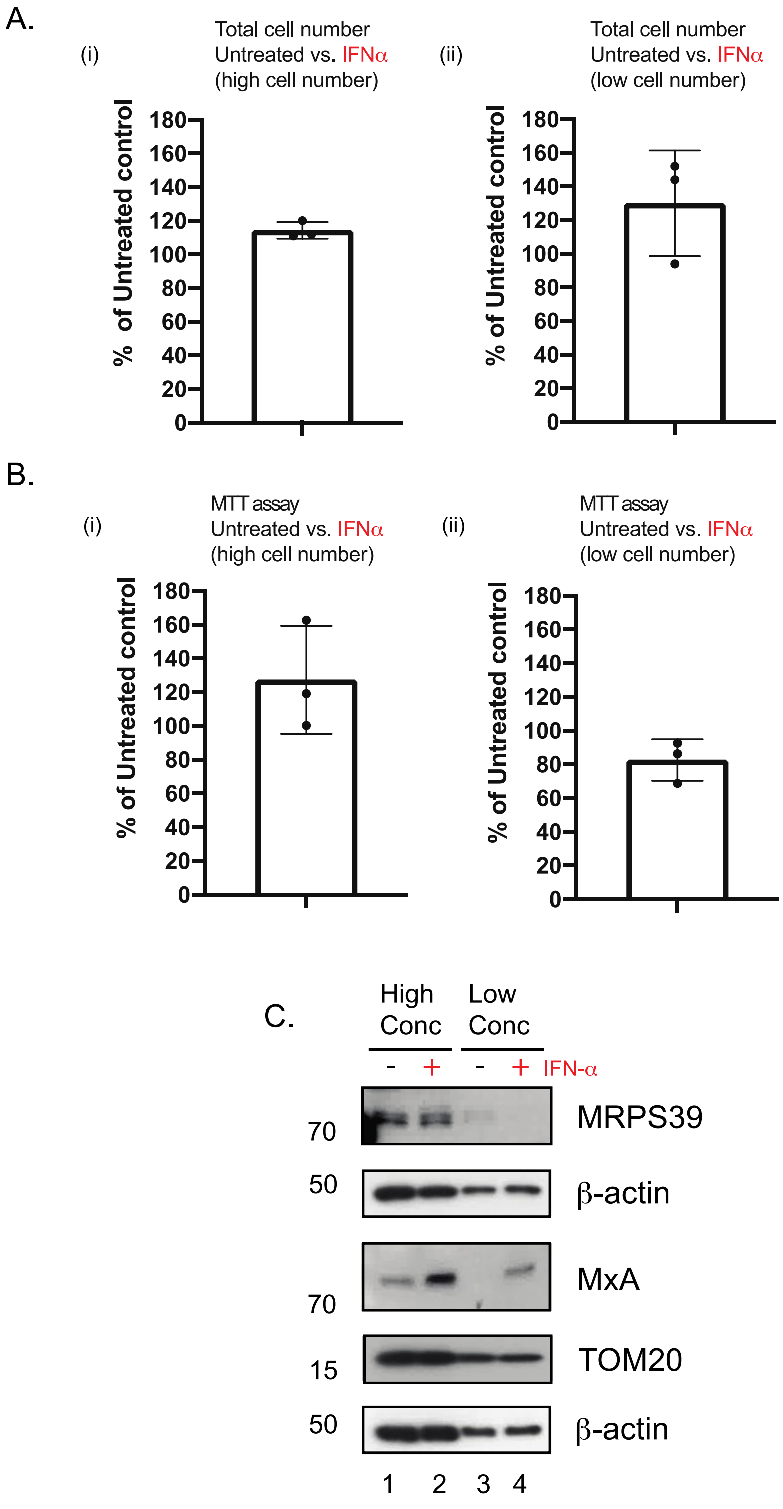
A.
(i)
Total cell number
Untreated vs. IFNα
(high cell number)
% of Untreated control
180
160
140
120
100
80
60
40
20
0
(ii)
Total cell number
Untreated vs. IFNα
(low cell number)
% of Untreated control
B.
(i)
MTT assay
Untreated vs. IFNα
(high cell number)
% of Untreated control
(ii)
MTT assay
Untreated vs. IFNα
(low cell number)
% of Untreated control
C.
High Conc
Low Conc
- + - + IFN-α
70
MRPS39
50
β-actin
MxA
70
TOM20
15
50
β-actin
1 2 3 4

Figure S2

Figure S3 HCMV replication in HFF cells treated with IFNα or IFNλ3. (A) (i) HFF cells were pre-treated for 24 hours with either IFNα, IFNλ3 or left untreated and then infected in the presence and absence of IFNα or IFNλ3. Treatment of cells with interferon proteins continued throughout infection with either AD169 or Merlin(R1111) for 96 hours. Titre in plaque forming units/ml (p.f.u./ml) of each experiment was calculated. Data is representative of three independent experiments (black data points) and presented as average (block) and standard deviation (error bars) of the data. Statistical relevance was examined used a student t test. ns = not significant (ns), p=<0.05 (*,**). (ii) and (iii) Fold decrease in HCMV titre in the presence of IFNα or IFNλ3, respectively, compared to HCMV titre from infected untreated cells. (B) Western blotting of uninfected HFF cells prepared at 24 hours post treatment with either IFNα or IFNλ3. Proteins recognized by the antibodies used in the experiment are indicated to the right of the figure. The positions of molecular weight markers (kDa) are indicated to the left of the figure.

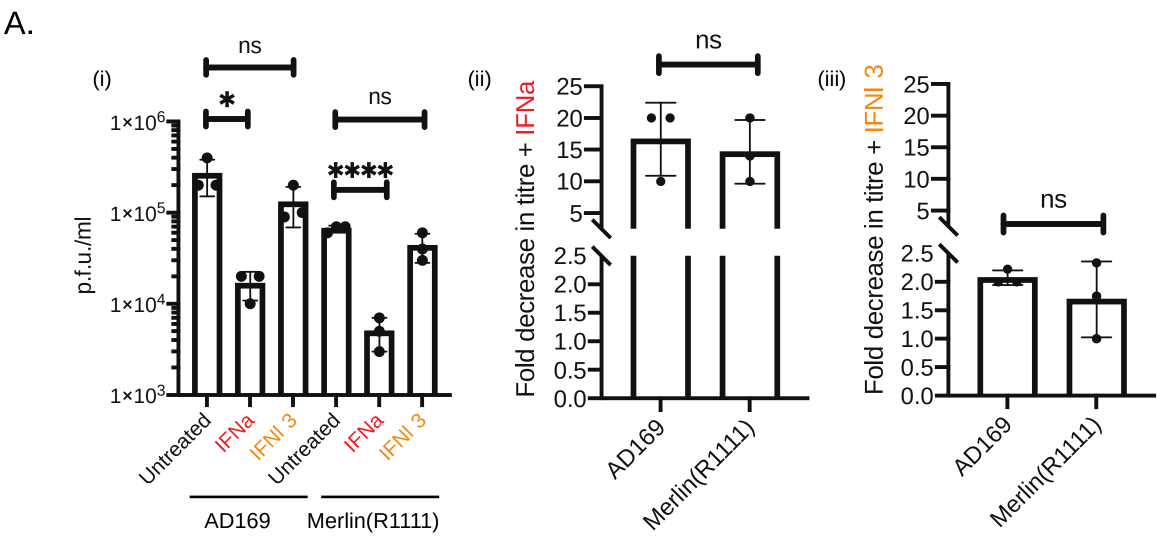
A.
(i)
ns
*
ns
****
1×10^6
1×10^5
1×10^4
1×10^3
p.f.u./ml
Untreated
IFNa
IFNl 3
Untreated
IFNa
IFNl 3
AD169
Merlin(R1111)
(ii)
Fold decrease in titre + IFNa
ns
AD169
Merlin(R1111)
(iii)
Fold decrease in titre + IFNl 3
ns
AD169
Merlin(R1111)

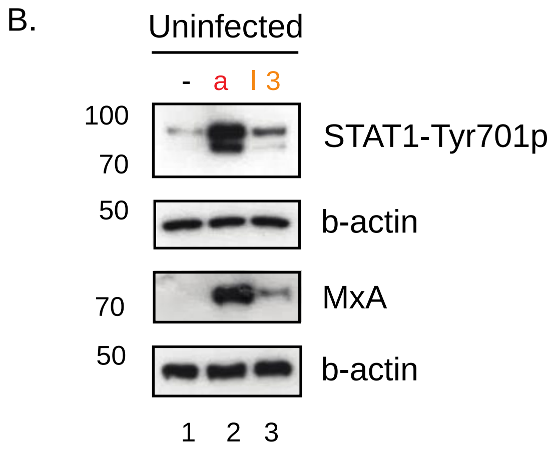
B.
Uninfected
- a l 3
100
70
STAT1-Tyr701p
50
b-actin
70
MxA
50
b-actin
1 2 3

Figure S3
